# Supplementary material for: A Shorter Form of the Work Extrinsic and Intrinsic Motivation Scale: Construction and Factorial Validation
Source: Int J Environ Res Public Health. 2022 Oct 25;19(21):13864. doi: 10.3390/ijerph192113864 (PMC9658934; doi:10.3390/ijerph192113864)

Boxplots demonstrating that no outliers were detected.

Intrinsic Motivation

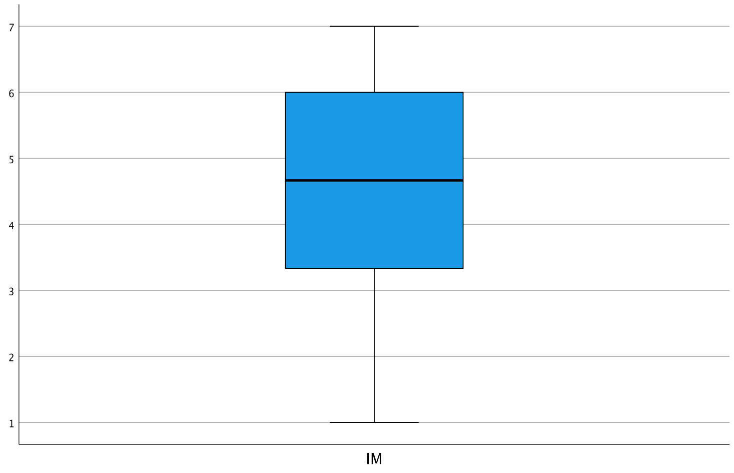

Integrated Regulation

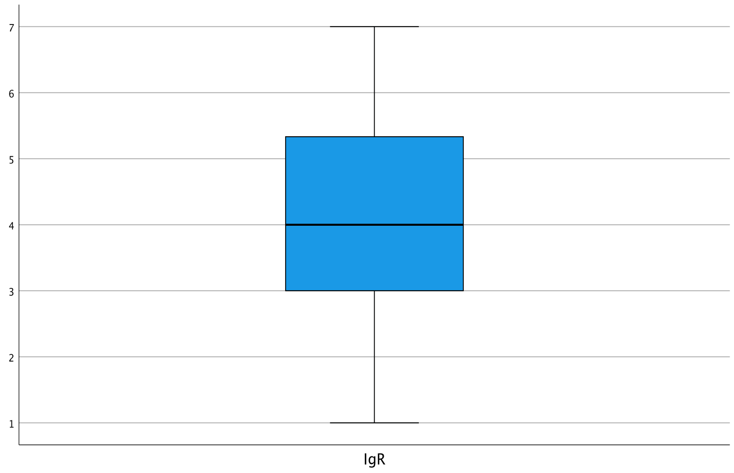

Identified Regulation

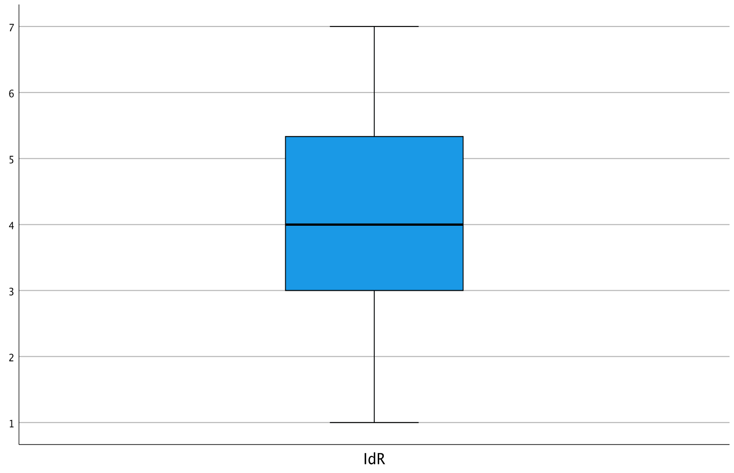

Introjected Regulation

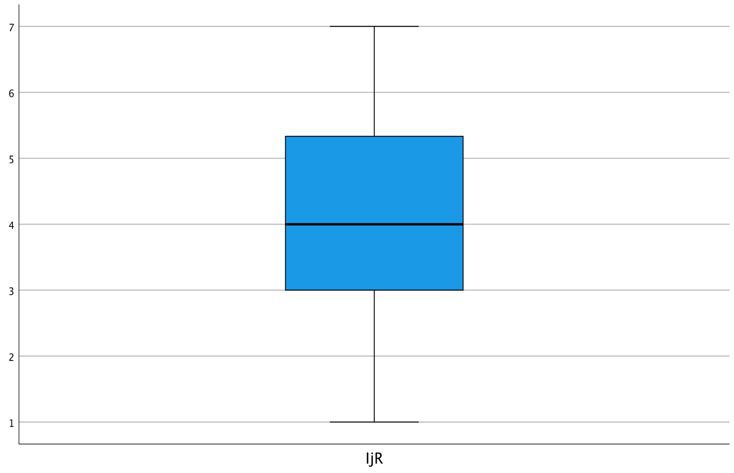

External Regulation

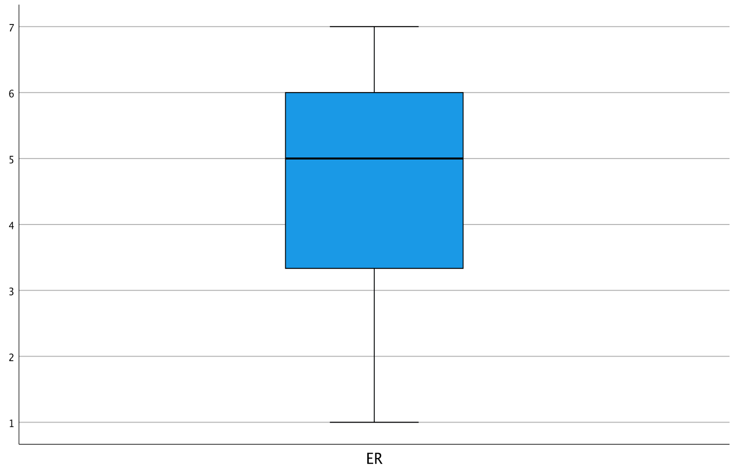

Amotivation

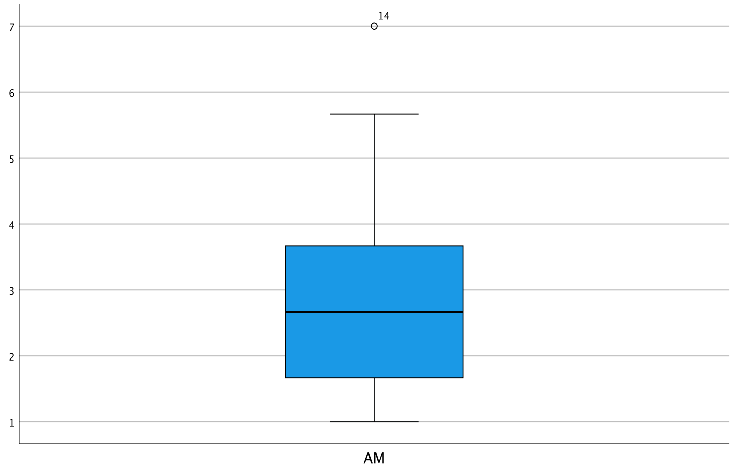

Supplement: Supplementary file 1 [file ijerph-19-13864-s001.zip › ijerph-1983075-supplementary.pdf]
